# Soluble CD14 as a Diagnostic Biomarker for Smear-Negative HIV-Associated Tuberculosis

**DOI:** 10.3390/pathogens7010026

**Published:** 2018-02-27

**Authors:** Yanyan Liu, Okechukwu C. Ndumnego, Tingting Chen, Ryung S. Kim, Elizabeth R. Jenny-Avital, Thumbi Ndung’u, Douglas Wilson, Jacqueline M. Achkar

**Affiliations:** 1 Department of Medicine, Albert Einstein College of Medicine, New York, NY 10461, USA; yanyan.liu@einstein.yu.edu (Y.L.); tingting.chen@einstein.yu.edu (T.C.); Elizabeth.Jenny-Avital@nbhn.net (E.R.J.-A.); 2 Africa Health Research Institute, Durban 4013, South Africa; okeyndumnego@gmail.com (O.C.N.); ndungu@ukzn.ac.za (T.N.); 3 Department of Epidemiology and Population Health, Albert Einstein College of Medicine, New York, NY 10461, USA; ryung.kim@einstein.yu.edu; 4 HIV Pathogenesis Programme, Nelson R. Mandela School of Medicine, University of KwaZulu-Natal, Durban 4013, South Africa; 5 Ragon Institute of MGH, MIT and Harvard University, Cambridge, MA 02139, USA; 6 Max Planck Institute of Infection Biology, 10117 Berlin, Germany; 7 Department of Medicine, Edendale Hospital, University of KwaZulu-Natal, Pietermaritzburg 3201, South Africa; wilsondpk@gmail.com; 8 Department of Microbiology and Immunology, Albert Einstein College of Medicine, New York, NY 10461, USA

**Keywords:** tuberculosis, biomarker, diagnostics, HIV, CD14, C-reactive protein

## Abstract

Sputum smear-negative HIV-associated active tuberculosis (TB) is challenging to diagnose. CD14 is a pattern recognition receptor that is known to mediate monocyte activation. Prior studies have shown increased levels of soluble CD14 (sCD14) as a potential biomarker for TB, but little is known about its value in detecting smear-negative HIV-associated TB. We optimized a sandwich ELISA for the detection of sCD14, and tested sera from 56 smear-negative South African (39 culture-positive and 17 culture-negative) HIV-infected pulmonary TB patients and 24 South African and 43 US (21 positive and 22 negative for tuberculin skin test, respectively) HIV-infected controls. SCD14 concentrations were significantly elevated in smear-negative HIV-associated TB compared with the HIV-infected controls (*p* < 0.0001), who had similar concentrations, irrespective of the country of origin or the presence or absence of latent *M. tuberculosis* infection (*p* = 0.19). The culture-confirmed TB group had a median sCD14 level of 2199 ng/mL (interquartile range 1927–2719 ng/mL), versus 1148 ng/mL (interquartile range 1053–1412 ng/mL) for the South African controls. At a specificity of 96%, sCD14 had a sensitivity of 95% for culture-confirmed smear-negative TB. These data indicate that sCD14 could be a highly accurate biomarker for the detection of HIV-associated TB.

## 1. Introduction

Active tuberculosis (TB) is the leading cause of death from a single infectious agent worldwide [1]. In 2016, an estimated 1.3 million and 0.4 million deaths were reported due to TB among HIV uninfected and co-infected persons, respectively [1]. It is postulated that a third of the world’s population is latently infected with *Mycobacterium tuberculosis* (*M. tuberculosis*), with around a 10% risk of developing TB within the first five years post-infection [2,3]. Co-infection with HIV increases the lifetime risk of developing active TB to about 30–60%, rising as host immunity deteriorates [4,5]. The diagnosis of HIV-associated TB is especially challenging since HIV co-infected patients form less organized granuloma and have a higher proportion of sputum smear-negative pulmonary TB as well as extra-pulmonary TB, especially at low CD4 counts [6,7,8,9]. Sputum microscopy for acid-fast bacilli (AFB), the most commonly used rapid point-of-care (POC) test for TB, typically has a sensitivity below 50%, with numbers reported to be as low as 10% for HIV-associated TB in some screening studies [10,11]. Gold standard TB diagnostics have higher sensitivities, but sputum or other body fluid cultures require laboratory infrastructure and weeks of incubation. Meanwhile, nucleic acid amplification (NAAT) tests such as the GeneXpert^®^ provide rapid results, but current versions are less sensitive than culture, and still require technology that is not available in all of the TB endemic resource-limited settings [12]. Even when available, sputum GeneXpert can have a limited clinical value in high HIV prevalence TB endemic settings, such as South Africa. For example, in a recent review of 222 patients receiving GeneXpert at a South African regional hospital, 61 (27%) were diagnosed with TB, but only 34 (56%) of those were GeneXpert-positive [13]. Thus, simple adjunctive tests that can facilitate triage testing to identify the HIV-infected TB suspects who would benefit from further confirmatory TB testing are needed [14].

Some simple adjunctive diagnostics or screening methods for HIV-associated TB are already available. These include the detection of the mycobacterial glycolipid lipoarabinomannan (LAM) in the urine by a lateral flow (dipstick) test. This very simple POC test has limited sensitivity in HIV uninfected TB patients, but in HIV-positive adults, it has a sensitivity and specificity of 56% and 90%, respectively, and has been endorsed by the World Health Organization (WHO) for the diagnosis of HIV-associated TB in patients with CD4 counts ≤100 cells/mm^3^ [15,16]. Another simple method is the measurement of C-reactive protein (CRP), an acute-phase protein that rapidly increases following the induction of inflammation due to infectious, autoimmune, or other inflammatory diseases [17,18,19,20,21]. As such, increased CRP levels are non-specific, but elevated levels have been documented in TB with or without HIV co-infection ([22,23,24,25,26,27] and reviewed by Yoon et al. 2017 [28]). Since CRP is already available as a rapid blood test in the clinical setting, it was found to be useful as a screening tool for HIV-associated TB in recent studies [22,24,26,27]. Wilson et al. utilized CRP in screening studies for smear-negative TB in symptomatic ambulatory patients in South Africa, and found a high sensitivity of 98%, albeit at low specificities of 51–59% [22,24]. 

Although currently not available as a routine test in clinical practice, the detection of CD14 could become another useful adjunctive method for screening for HIV-associated TB. CD14 is a pattern recognition receptor that is known to bind numerous microbial products and mediate monocyte activation [29,30,31,32]. It is expressed as a receptor on the surface of monocytes and polymorphonuclear cells [33,34], and in soluble forms (sCD14) in the blood following monocyte activation and shedding [35,36]. The engagement of CD14 by bacterial lipopolysaccharide (LPS) and cell wall components induces cellular activation and the secretion of inflammatory cytokines [37,38]. Increased plasma levels of sCD14 have been documented in TB patients both with and without HIV co-infection, compared to those with latent or no *M. tuberculosis* infection [39,40,41,42]. More recently, we identified sCD14 among a subset of host protein biomarkers that are able to distinguish TB from other respiratory diseases with high accuracy in both HIV-infected and uninfected subjects [43]. It thus could be a more specific inflammatory marker than CRP. However, little is known about the potential value of sCD14 as a diagnostic biomarker for smear-negative HIV-associated TB, the disease form that is most challenging to diagnose. We hypothesized that sCD14 can serve as a diagnostic aid in screening for smear-negative HIV-associated TB. Our objectives were to (i) evaluate the sensitivity and specificity of sCD14 for smear-negative HIV-associated TB; and (ii) to correlate sCD14 and CRP values.

## 2. Results

### 2.1. Soluble CD14 Serum Concentrations with Sensitivity and Specificity for Smear-Negative HIV-Associated TB

**Figure 1:**
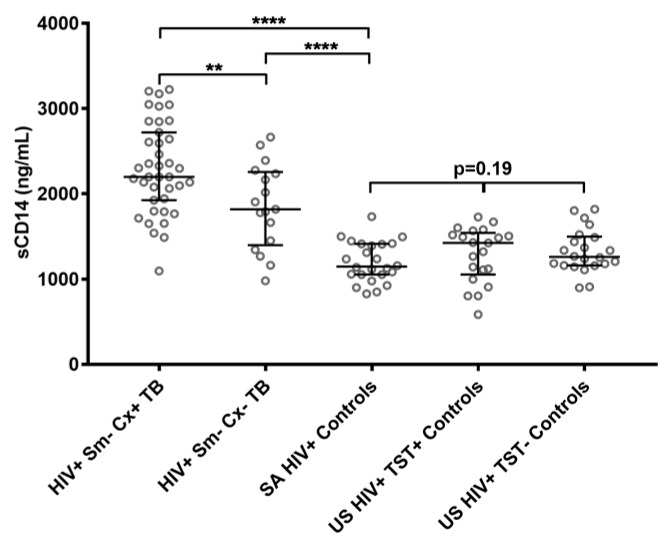
Soluble CD14 (sCD14) values of HIV-infected subjects with smear-negative culture-confirmed TB (HIV+ Sm- Cx+ TB; *n* = 39), smear-negative culture-negative TB (HIV+ Sm- Cx- TB; *n* = 17), South African HIV-infected controls (SA HIV+ Controls; *n* = 24), US HIV-infected Tuberculin skin-test (TST) positive (US HIV+ TST+; *n* = 21), and US HIV-infected TST-negative (US HIV+ TST-; *n* = 22) controls. Bars show median sCD14 values with interquartile ranges. The Mann–Whitney *U* test was used for two-group comparisons, and the Kruskal–Wallis test was used for multiple-group comparisons. * *p* < 0.05, ** *p* < 0.01, *** *p* < 0.001, and **** *p* < 0.0001.

**Figure 2:**
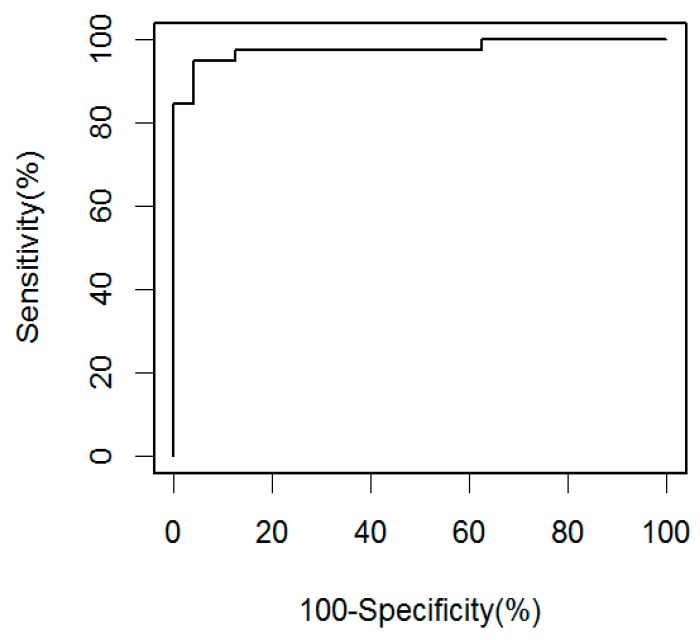
Receiver operation characteristics (ROC) curve based on sCD14 concentrations of HIV-infected smear-negative culture-confirmed TB patients (*n* = 39), and South African HIV-infected controls (*n* = 24).

Soluble CD14 levels were measured in sera from 56 South African smear-negative (39 culture-positive and 17 culture-negative) HIV-infected pulmonary TB patients (Table 1), and 24 South African and 43 United States (US) HIV-infected controls (21 tuberculin skin test (TST)-positive and 22 TST-negative; Table 2). Results were reproducible, and correlated highly between assays performed on two separate days (r = 0.79; *p* < 0.0001). The sCD14 levels of the South African smear-negative culture-confirmed HIV-infected TB patients had a median concentration of 2199 ng/mL (interquartile range 1927–2719 ng/mL), and were significantly elevated compared to the South African controls, who had a median of 1148 ng/mL (interquartile range 1053–1412 ng/mL; *p* < 0.0001; Figure 1). There was no significant difference in sCD14 levels among the HIV-infected control groups (*p* = 0.19), and in two group comparisons between the South African and US (*p* = 0.07), and between TST+ and TST- controls (*p* = 0.89). However, higher sCD14 levels were detected in culture-confirmed patients compared with culture-negative TB patients (*p* < 0.01). Although culture-negative TB patients responded clinically to antituberculous treatment, we could not be absolutely certain that all of them had definite TB. Therefore, our receiver operating characteristic (ROC) curve analysis was based on HIV-infected smear-negative culture-confirmed TB patients and South African HIV-infected controls enrolled in the same setting (Figure 2). At a cutoff of 1540 ng/mL, the sensitivity for smear-negative culture-confirmed TB was 95% with a specificity of 96%. When keeping that same cutoff value, the sensitivity for culture-negative TB would be 71%.

### 2.2. Correlations of sCD14 and CD4 Levels

**Figure 3:**
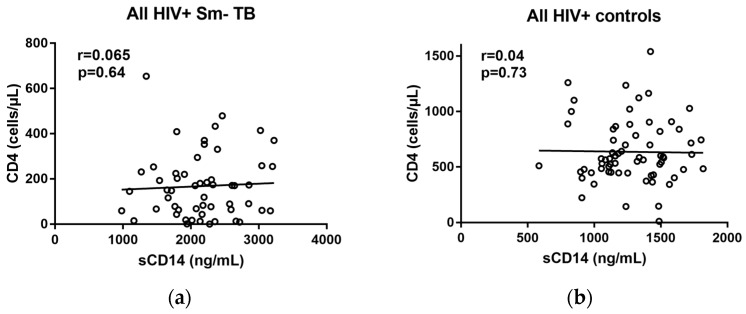
Correlations between sCD14 and CD4 levels in all of the South African HIV+ Sm- TB cases (**a**), and all of the controls from South Africa and the United States (**b**). Spearmen rank correlation.

To assure that the clinical value of sCD14 as an HIV-associated TB biomarker was not influenced by level of immunosuppression, we correlated sCD14 levels with CD4 counts in TB patients and controls. In all of the groups, sCD14 levels did not correlate significantly with CD4 counts (Figure 3).

### 2.3. Correlations of sCD14 and CRP Levels

**Figure 4:**
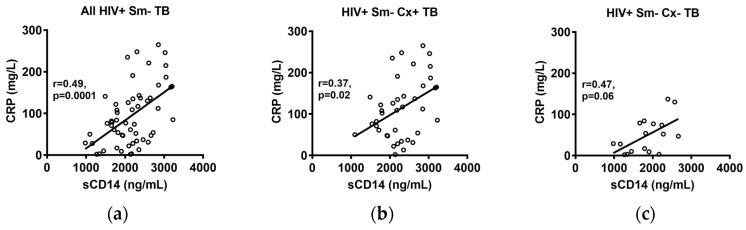
Correlations between sCD14 levels and C-reactive protein (CRP) values in all of the South African HIV+ Sm- TB cases (**a**), HIV+ Sm- Cx+ TB cases (**b**) and HIV+ Sm- Cx- TB cases (**c**). Spearmen rank correlation.

The concentrations of CRP were available for the South African smear-negative TB cases [24]. A moderate but highly significant (r = 0.49, *p* = 0.0001) correlation was observed between sCD14 levels and the CRP levels in all of the TB cases combined (Figure 4a), with similar correlation values for the culture-confirmed and culture-negative TB cases (Figure 4b,c).

## 3. Discussion

Our study contributes important data towards the clinical value of sCD14 as an accurate biomarker for HIV-associated TB. While our results are consistent with prior studies showing significantly elevated sCD14 in HIV-associated TB compared with asymptomatic HIV+ *M. tuberculosis*-infected and uninfected controls [39,40,41], we further demonstrate that sCD14 has both a high sensitivity (96%) and specificity (95%) for the detection of smear-negative culture-confirmed HIV-associated TB. These findings are clinically relevant, because (i) the rate of smear-negative TB has increased in countries with a high HIV prevalence [45,46]; (ii) smear-negative TB is often very challenging to diagnose in HIV-infected individuals, in whom the differential diagnoses of other opportunistic diseases with similar signs and symptoms can be quite large; and (iii) affected persons have a higher risk of death than patients with smear-positive TB [46,47,48]. Furthermore, measurements of sCD14 can be adapted to a simple lateral flow format that is similar to those already available for the detection of other host-derived proteins such as CRP [49], prostate-specific antigen [50], human chorionic gonadotropin [51], and D-dimer [52]. Since the lateral flow format has a similar immunological recognition principle as the sandwich immunoassay used in our study, it would have a similar detection profile, but obviate the need for laboratory facilities and specialist training [53,54]. It could thus serve as a non-sputum-based POC test for HIV-associated TB that exceeds the WHO and Centers for Disease Control and Prevention (CDC) target product profile of at least 90% sensitivity with 70–80% specificity, and is usable in resource-limited settings and at the level of community-based health centers [14].

At an optimal cutoff calculated for smear-negative culture-confirmed TB, sCD14 had a lower, but nevertheless considerably high sensitivity for culture-negative TB (71%), indicating that sCD14 could detect this most difficult to diagnose form of HIV-associated TB. It is conceivable that the lower sCD14 in the culture-negative TB patients could have been due to a lower inflammatory response in a group that appeared to be more immunocompromised, and had a median of 68 cells/mm^3^; this was a lower, though not significantly different CD4 count than the smear-negative culture-confirmed group with a median of 119 cells/mm^3^. Another explanation could be that although all of the patients responded clinically to antituberculous treatment, not all of them truly had TB. Larger studies with additional diagnostic assessments are needed in order to validate the value of sCD14 as a biomarker for culture-negative HIV-associated TB.

Monocytes and macrophages, the main producers of sCD14, also harbor HIV [36,55,56,57,58]. It has long been known that sCD14 is also a biomarker for HIV-associated immune activation because of HIV-associated intestinal mucosa disruption and consequent microbial product translocation such as LPSs triggering inflammation and immune activation, which includes monocyte activation [59,60,61,62,63]. We did not find a significant correlation between sCD14 levels and CD4 counts, neither in the TB patients nor in the controls, indicating that sCD14 concentrations are not additionally influenced by the level of immunosuppression in *M. tuberculosis* HIV co-infected individuals. This was an important observation, as it suggests that, in contrast to the detection of urinary LAM, the TB biomarker value of sCD14 is not limited to a patient group with certain CD14 values. However, our findings were inconsistent with the observations by Sullivan et al., who found a significantly negative correlation between sCD14 and CD4 counts when combining data from South African HIV-infected *M. tuberculosis* uninfected, latent tuberculosis infection (LTBI), TB patients in their analysis [40]. The lack of a significant correlation between sCD14 and CD4 in our HIV-infected controls from both the US and South Africa was also inconsistent with data from some, but not all, of the studies that investigated correlations between sCD14 levels and CD4 counts in the context of HIV progression [63,64,65]. Studies that found a significant negative correlation between sCD14 and CD4 reported r values ranging from −0.66–−0.41, while others found a weak (r = −0.17) or no significant correlation [62,66,67]. Additional studies are thus needed to investigate whether there could be an association between sC14 and CD4 in the setting of HIV *M. tuberculosis* co-infection.

With sensitivities reported up to 98%, serum CRP values have been found to be useful in the screening for TB in high HIV prevalence settings [22,24,26,27]. Since the CRP concentrations for the South African smear-negative cases in this study were available from a previous study [24], we compared these to the sCD14 levels in the same subjects, and found a moderate but highly significant correlation (r = 0.49, *p* = 0.0001). While this correlation is not surprising, given the immune activation in the TB patients, its moderate value raises the question of whether sCD14 and CRP could have a complementary value in the screening or triaging for TB in HIV-infected persons. On the other hand, it is important to note that sCD14 appears to have a much higher specificity than the 51–59% reported for CRP [22,24], and thus a combination of both biomarkers would likely result in a decreased specificity, driven by the lack of specificity of CRP.

Although we were able to obtain samples from a large group of smear-negative HIV-associated TB patients, our study was limited by the analysis of samples from South African TB patients, and by the lack of availability of samples from patients with other confirmed HIV-associated diseases. However, using mass spectrometry analysis, we recently reported that sCD14 was among a few host proteins from a small biomarker panel that could distinguish HIV-associated TB from other HIV-associated respiratory diseases with high accuracy (area under the curve (AUC) of 0.95) [43]. Since the samples for this study came from patients who were born in different TB endemic regions of various continents and had immigrated to the US, we hypothesize that sCD14 can distinguish between TB and other respiratory diseases, regardless of setting. Larger multinational studies are needed to prove this hypothesis.

In summary, our results support prior work, and further demonstrate that sCD14 is a highly accurate biomarker for smear-negative HIV-associated TB. They suggest a role for sCD14 in the early screening or triaging for active TB in HIV-infected subjects in order to identify those individuals who should undergo further TB confirmatory testing. Our results further posit for a role of sCD14 measurements as a diagnostic tool for HIV-associated TB.

## 4. Materials and Methods 

### 4.1. Study Design and Subjects

This study utilized samples from a prospectively performed study with a consecutive enrollment of South African HIV-infected patients suspected of having TB [24]. These samples have previously been tested for CRP and the antibody biomarker for HIV-associated TB [22,44]. Briefly, samples were obtained from symptomatic (cough >two weeks) South African HIV-infected adults (≥18 years old) who were either suspected of having TB (Table 1), or from South African and US HIV-infected asymptomatic adult controls (Table 2). Consecutive symptomatic South African patients with negative sputum microscopy (two sputum smears) were referred from primary care facilities and then enrolled at Edendale Hospital, a township hospital in the South African province KwaZulu-Natal, and followed for eight weeks [24]. Patients who took any antituberculous drugs within the three months prior to enrollment were excluded. According to South African guidelines, all of the smear-negative TB subjects had received a five-day course of antibiotics (amoxicillin/clavulanic acid or doxycycline) without clinical improvement prior to enrollment. Sera from patients who were ultimately diagnosed with sputum smear-negative TB, which was either confirmed by a positive sputum culture for *M. tuberculosis* (*n* = 39) several weeks later, or, if culture negative, by a clinical response to antituberculous drug treatment after eight weeks (*n* = 17), were tested in this study, and compared with sera from adult controls. Of note, regardless of culture positivity, 69% of TB patients had radiographic abnormalities that were consistent with TB on chest X-ray. South African control subjects were enrolled at an HIV outpatient clinic at Edendale Hospital, and US controls were enrolled at an HIV outpatient clinic at Jacobi Hospital, Bronx, NY. While South African controls had no specific tests performed aside from HIV and CD4 testing, all of the US controls had a normal chest X-ray, and a recorded result for a prior TST, which was considered positive if the induration was ≥5mm [68]. Routine TST (or radiologic imaging) is typically not performed in South Africa, due to BCG vaccination at birth and high rates of latent *M. tuberculosis* infection. Since subclinical TB, a form of asymptomatic TB, has been reported in up to 10% of HIV-associated TB cases in TB-endemic regions (reviewed by Achkar & Jenny-Avital 2011 [6]), we enrolled South African controls with CD4 ≥300 cells/mm^3^ in order to reduce the likelihood of undetected subclinical TB. Exclusion criteria for controls were (i) a history of treated TB within the year prior to enrollment, and (ii) symptoms compatible with TB. Blood was obtained after subjects provided written informed consent; it was processed and aliquoted on the same day, and stored at −80 °C until tested. Demographic and clinical information was obtained from interviews and medical records. Approval for human subject research was obtained from the Biomedical Research Ethics Committees of the University of KwaZulu-Natal, the KwaZulu-Natal Department of Health, and the Institutional Review Board of the Albert Einstein College of Medicine.

### 4.2. Sample Preparation

Sera were thawed on the day of the experiments. HIV was inactivated by incubating sera with an equal volume of 2% Triton X-100 for 30 min at room temperature [69], followed by serum dilution (1:600) in 1% bovine serum albumin (BSA) in phosphate-buffered saline (PBS).

### 4.3. ELISAs

Soluble CD14 was measured using a commercially available sandwich ELISA kit (Human CD14 DuoSet ELISA, DY383, R&D systems, Minneapolis, MN, USA) after optimizing the manufacturer’s protocol for the assessment of sCD14 concentrations. Briefly, the wells of 96-well microtiter plates (Costar, 9018, Corning, Corning, NY, USA) were coated with 100 µL of sCD14 capture antibody at 2 µg/mL, and incubated overnight at 25 °C. After washing with 0.05% Tween 20 in PBS, plates were blocked with 1% BSA in PBS, and incubated at 25 °C for 1 h. After further washing, diluted serum samples (1:600) were added at 100 µL per well, and incubated at 25 °C for 1.5 h. Plates were then washed, and 100 µL of sCD14 detection antibody (75 ng/mL) was added to each well. Then, the plates were incubated for 1.5 h at 25 °C, washed again, and 100 µL of Streptavidin-HRP (1:200 in dilution buffer) was added per well, which was followed by incubation for 20 min at 25 °C. After another washing, there was the addition of substrate solution (Substrate Reagent Pack, DY999, R&D systems, Minneapolis, MN, USA), incubation at 25 °C for 20 min, and the addition of stop solution (2 N sulfuric acid). Then, the optical densities (OD) were determined by subtracting the OD at 530 nm from 450 nm to correct the optical imperfections of the plate, as per the manufacturer’s instructions, using a microplate reader (Spectra MAX, Molecular Devices, San Jose, CA, USA). Human sCD14 standards (Sigma-Aldrich, St. Louis, MO, USA; serially diluted starting at 45 ng/mL,) were performed in parallel on all of the plates, and concentrations were calculated based on the generated standard curve. All of the plates included negative controls, which contained all of the components except for sera, for which dilution buffer was added instead. Positive controls consisted of the same two reference sera from two healthy volunteers for whom sCD14 concentrations were known (one high and one low sCD14 concentration), which were included as interplate controls on all of the assays. All of the assays were repeated on two separate days.

### 4.4. CRP Measurements

High-sensitivity CRP concentrations were determined in the parent study using a Dimension RXL analyzer from Dade-Behring (Deerfield, IL, USA) [22].

### 4.5. Statistical Analysis

Prism software, version 7 (GraphPad Software, Inc., San Diego, CA, USA) and R statistical software [70] were used for statistical analysis. Non-parametric tests were used—the Mann–Whitney *U* test for the two-group comparisons, the Kruskal–Wallis test for multiple-group comparisons, and the Spearmen rank test for correlations between sCD14 and CD4 counts or CRP values. The WHO, CDC, and other major TB stakeholders recently recommended a sensitivity of at least 90% with a specificity of 70–80% for a non-sputum-based diagnostic POC TB [14]. We therefore chose a cutoff for a positive sCD14 value based on ROC curve analysis, with the objective of obtaining over 90% (ideally 95%) sensitivity with at least 80% specificity using values from the smear-negative culture-confirmed South African TB patients and South African controls.

## Figures and Tables

**Table 1:** Demographics and clinical characteristics of smear-negative HIV+ pulmonary-tuberculosis (TB) patients *.

Characteristic	Culture-Positive Subjects with TB (*n* = 39)	Culture-Negative Subjects with TB (*n* = 17)	*p* Value
Male sex (%)	19 (49)	8 (47)	0.91 ^a^
Age, mean years (±SD)	33 (±7)	32 (±6)	0.86 ^b^
CD4 cells/mm^3^, median (IQR) ^d^	119 (18–219)	68 (15–193)	0.41 ^c^
CRP mg/L, median (IQR)	109 (52–164)	47 (10–77)	0.001 ^c^
History of prior TB (%)	8 (21)	1 (6)	0.25 ^b^

* adapted from [24,44]; ^a^ Chi-square test; ^b^ *t* test; ^c^ Mann–Whitney *U* test; ^d^ IQR, interquartile range.

**Table 2:** Characteristics of control subjects *.

Characteristic	US HIV+ TST− (*n* = 22)	US HIV+ TST+ (*n* = 21)	SA HIV+ (*n* = 24)
Age, mean no. of years (±SD)	48 (±10)	46 (±13)	33 (±7)
Male sex (%)	10 (46)	14 (64)	6 (25)
CD4 cells/mm^3^, median (IQR)	574 (449–744)	525 (423–842)	601 (483–742)

* adapted from [44]; US: United States; SA: South African.
